# The healthy eating movement on social media and its psychological effects on body image

**DOI:** 10.3389/fnut.2024.1474729

**Published:** 2024-12-17

**Authors:** Alina Zaharia, Iulia Gonța

**Affiliations:** ^1^Department of Psychology and Psychotherapy, Faculty of Psychology, Titu Maiorescu University, Bucharest, Romania; ^2^Teacher Training and Social Sciences Department, National University of Science and Technology POLITEHNICA Bucharest, Bucharest, Romania

**Keywords:** social media pressure, body image, emotional eating behavior, thin body, beauty standards, thinspiration, fitspiration

## Abstract

**Introduction:**

The present study aims to investigate the relationship between social-media pressure, the tendency to internalize standards of beauty and attractiveness associated with thin bodies, which subsequently leads to distortion of body shape perceptions, and restrictive and emotional eating behavior disorders.

**Methods:**

A survey-based research design was employed, utilizing an online questionnaire to collect data. The study sample consisted of 614 students, selected from the most prestigious universities in Bucharest. The questionnaire incorporated validated scales measuring Socio-Media Pressure for a Thin Body Image (SMPTB), Body Appreciation (BA), Body Shape Perception (BSP), Restrained Eating Behavior (REB), and Emotional Eating Behavior (EEB).

**Results:**

The results confirmed the hypotheses of the research, meaning food restrictions are a way to diminish the level of dissatisfaction with body shape, to reduce the difference between the ideal body shape and the real one. Food restrictions are perceived as natural behaviors, appropriate to support the standards of beauty and attractiveness specific to this historical stage. Emotional eating disorders emerge as a way to compensate for the discomfort generated by low body esteem.

**Discussion:**

The results underscore the pervasive influence of social media in shaping eating behaviors and body image perceptions. Food restrictions, framed as natural responses to societal pressures, highlight the need for interventions addressing the normalization of harmful beauty standards. Emotional eating behaviors reveal the psychological toll of body dissatisfaction, emphasizing the importance of strategies to foster positive body image and mental well-being. These findings provide a foundation for developing educational campaigns and therapeutic approaches targeting the psychological impact of social media on eating behaviors.

## 1 Introduction

In contemporary society, we are witnessing an increasing tendency in people to function, relate, work using technology, build benchmarks and follow models promoted on social networks. Also, individuals tend more and more to rely on information provided by different modern means of communication at the level of their social groups (1). Covid-19 pandemic has created a favorable context for the intensive use of technology in order to maintain interpersonal relationships, to have entertainment and relaxation, and to carry out professional activities (2–5). The cost that people pay in order to ease their work, to get informed quickly or to relax refer to the internalization of the messages underlying the content of social media. A significant category of these messages refers to aspects related to the need of having a slim body (6, 7), or adopting eating behaviors conducive to these standards. Exposure to images of thin bodies, bodies worked in the gym, does not produce the expected effects: adopting a healthy lifestyle, involving sports, movement, appropriate eating behaviors, but on the contrary, deepens the discordance between the desired body image, inoculated through social media, and the real image. The constant exposure to idealized body images on social media platforms can lead to increased body dissatisfaction and a low level of self-esteem among users. Studies have shown that this phenomenon is particularly prevalent among adolescents and young adults, who are more susceptible to peer influence and social comparison (8, 9). Moreover, the pressure to conform to these unrealistic body standards can result in the adoption of unhealthy behaviors, such as extreme dieting, over-exercising, and even eating disorders (10, 11). These behaviors are often fueled by the desire to achieve the “perfect” body image that is frequently portrayed on social media. Interestingly, while social media can have detrimental effects on body image and behavior, it also has the potential to promote positive health behaviors. For instance, some social media campaigns and influencers advocate for body positivity and healthy living, which can inspire individuals to adopt healthier lifestyles (12, 13). Furthermore, the use of social media for health promotion is becoming increasingly popular. Health professionals and organizations leverage social media platforms to disseminate information about healthy eating, physical activity, and mental well-being, aiming to counteract the negative effects of exposure to idealized body images (14, 15). It is crucial for individuals to develop critical media literacy skills to navigate the complex landscape of social media. By understanding the constructed nature of the content they consume, individuals can better resist the pressure to conform to unrealistic body standards and make more informed decisions about their health and well-being (9, 16). In the light of the above considerations, the research data brought by this study is useful for understanding the issues associated with social reflection mechanisms, relevant in the approach of building perceptions of body form (17, 18), in order to outline educational strategies for children, adolescents and young people, customized, depending on the age category, but also the specifics of the media content. The present study aims to present the psychological implications of association relationships between social-media pressure regarding ideal body image, internalization of a standard of beauty and attractiveness of thin bodies, quality of body shape perceptions, level of body appreciation, and the presence of restrictive and emotional eating behavior disorders. The paper is structured as follows: introduction makes a brief presentation of the paper emphasizing the novelty and the methodology of research, second part presents a literature review designed to substantiate the six working hypotheses and to propose our theoretical model. The third part presents the research methodology and materials, followed by the fourth part witch, presents the research findings to the readers. The fifth and sixth parts are revealing the discussions and conclusions of the paper, pointing out the main ideas and contributions in the field.

## 2 Literature review and hypotheses development

The present study aims to present the psychological implications of the body image construction approach, considering the aspects associated with the pressure to display an appearance that corresponds to the beauty model, specific to the era, promoted on social media platforms. This leads to the internalization of a standard of beauty associated with thin bodies and favors the tendency to evaluate and appreciate one’s own body based on comparison with thin and attractive bodies exhibited on social media. The consequences of these actions are materialized in the adoption of problematic eating behaviors.

In order to conceptualize these relations, we may focus our attention on the possible correlations between the high level of social media pressure and the tendency to internalize thin bodies standards. Social media platforms are powerful agents of influence in the process of building body image, with simple exposure to images and videos of thin, attractive bodies easily leading to the assimilation of beauty standards (19). The feeling of this influence by consumers can be defined as social media pressure. Young women prefer Facebook and Instagram to traditional forms of media such as television or magazines (20). This preference is explained by the nature of the content found on these platforms with some of them (e.g., Instagram) that allow photo and video editing (21), having also a high degree of addictiveness. Dependence on SNS (social networking service) is supported by multiple research data (22–25), studies that report the psychological, social and behavioral implications of social media content consumption, focusing mostly on the negative effects, through the unrealistic standards imposed by social media platforms regarding appearance. To highlight the impact of social media, a new concept is outlined, the construct of appearance-related social media consciousness, described “as the extent to which individuals’ thoughts and behaviors reflect ongoing awareness of whether they might look attractive to a social media audience” (26, p. 164). Thus, the analysis of one’s own body starts from what could be considered attractive in the social media space, from the physiological aspects validated at the social level. The tendency to internalize standards associated with thin bodies requires acceptance of socially valid models of beauty and attractiveness and pressure to follow those standards. SMPTB (27). In a study conducted on 259 women, aged between 18 and 29, it was observed that preoccupation with social media content related to appearance, materialized by activity involving photos on Facebook and Instagram, was associated with concerns about body image, the internalization of an ideal of the slim body that intensifies the desire for a slim body, aspects that were not found in the case of those who did not show interest in the issue of appearance on social media (28). Internalizing a standard of a slim body, following exposure to social media content related to appearance, and installing the need to follow that standard, are reported also in recent studies (29, 30). In another study, 193 college students, aged 17 to 46, were exposed to conventional images associated with the ideal of beauty by using Facebook, in order to highlight, the role, that social media platforms, play in comparing appearance, body image quality and intensity of eating disorders. Compared to the control group, people in the experimental group showed higher values in terms of the tendency to relate to images on social media, consequently an increase in the tendency to compare, which favors the degree of body image deformation and higher chances of adopting eating disorders (31). The intensification of the comparison based on appearance involves a strong internalization of social media content, which becomes a benchmark for how one’s own body is perceived and analyzed. The intense activity of sharing pictures of one’s own body on social networks, frequent especially among adolescent girls, is associated with greater control for weight and body shape, which is based on the internalization of a standard of beauty associated with thin bodies triggered by the use of social media platforms (32). Another study of 103 teenage girls reported results along the same lines. Adolescent girls were tested in terms of using the Facebook platform, aspects related to internalizing a slim body standard, appearance comparison, desire for a slim body, weight problems, tendency to be noticed and evaluated based on appearance, research data indicating significant correlations between psychological variables (33). The strong association between social media consumption and pressure to follow a pattern of ideal appearance is shown in a study (34) aimed at investigating 1,552 Norwegian adolescents, 827 girls and 725 boys. Adolescents reported consistent exposure to social media, most of them spending more than 2–3 h in the media space (28.5% – 2–3 h, 25.1% – 3–4 h, 25.3%–over 4 h). In terms of gender differences, girls spend much more time on social media space (30.7%–over 4 h) compared to boys (19.0%–over 4 h), which led to differences in other variables as well, so girls were more susceptible to social media pressure to adopt a standard associated with slim and attractive bodies and to internalize this model. Male adolescents showed higher averages only on the variable associated with the tendency to internalize a muscle body standard, which is explained by the presence of a greater number of images and videos on social media showing the bodies of men with muscles. The power that social media holds in creating trends related to physical appearance is illustrated by the trends of this historical milestone –“thinspiration and fitspiration” (7). Thinspiration involves presenting motivating messages and images that support lean bodies, and fitspiration supports the association between physical activity, health, and attractive bodies (35). Although representatives of these movements presented and promoted them in order to inoculate healthy body image cues, many studies report the opposite, creating the context for internalizing unhealthy patterns of beauty (36). These movements are defined as “classes of social media content characterized by idealized depictions of excessively thin and overtly fit/lean bodies” (37, p. 187). To investigate the effects that thinspiration and fitspiration, can generate in real life, 108 participants were asked to use their smartphones for a week to track measurements of them as well as body satisfaction. Women reported a considerably higher number of exposures to both material-related materials Thinspiration and Fitspiration, the average duration of exposure being 2–3 min, with no differences reported between the two content categories (38). Exposure to thinspiration and fitspiration content was associated with lower body satisfaction and overall functioning in both women and men. Thinspiration and Fitspiration are contemporary ways in which social media builds and promotes standard beauty images. In this regard, a descriptive study, which was based on the content analysis of images that were appreciated as a source of inspiration in the social media space, being considered three social networks: Instagram, Twitter and WeHeartIt, given their popularity, as well as the possibility of sharing photos (39). The study introduced into the analysis, in addition to thinspiration (promoting slim bodies with little body fat) and fitspiration (promoting toned bodies), a new construct—Bonespiration, movement similar to thinspiration, that promotes extremely thin bodies, whose bones are pronounced. The results illustrate significant differences in Thinspiration and Bonespiration content compared to Fitspiration content, with many more thin bodies present in the first two categories than in the third category. Fitspiration is perceived as the inspirational variant in social media that leads to the creation of healthier landmarks in terms of appearance, although some participants have associated Thinspiration with Fitspiration, which draws attention to the psychological and behavioral risks that can occur under the motto of messages such as Fitspiration. The aspects highlighted above, allow us to formulate the first hypothesis of our study (H1).

*H1:* A high level of social media pressure that individuals feel about ideal body image is associated with a high level of tendency to internalize the standard of beauty and attractiveness of slim bodies.

Considering the role that social media platforms play in shaping the perception of what constitutes an attractive body, we present aspects associated with the process of self-image formation specific to the current period.

Self-image includes a combination of perceptions, attitudes and impressions about oneself (40). Self-image is a construct that can take on positive or negative valences (41). A significant component of self-image is body image, especially at this historical stage, where we identify a growing pressure to follow the benchmarks promoted on social media, associated with thin, athletic bodies, models of beauty and attractiveness (42–45). Social media content is a powerful agent of influence in shaping the ideal of beauty. Body image illustrates the subjective perception of physical aspects, which is based on self-evaluation and social reflection (46). On the one hand, we identify the role of internal factors, such as personality structure and personal experiences, as well as external social factors, on the other hand. Family, groups of friends, and social media constitute a consistent facet of how a person perceives themselves (7). Forming a positive body image involves acceptance and respect for appearance. People with a positive self-image accept their body as it is, regardless of its shape. Thus, there is a differentiation between satisfaction with one’s own body and the perception formed in this sense (47). Dissatisfaction with one’s own body sets in when people describe their body in negative terms and there is a disharmony between real and ideal body image (48). Body shape perception distortion not only refers to a deformation of body perception, but also includes body-associated attitude and cognitive impairment (49). Thus, people who have sketched a distorted perception of their appearance, relate inappropriately to themselves, and emit thoughts of denigration towards themselves. It is not only a matter of measuring body shape perception, but also of self-discredit. The tendency to internalize a standard of beauty and attractiveness of thin bodies (TBISI—Thin Body Image Standard Internalization), a concept presented in the previous section, starting from unrealistic benchmarks in social media, will considerably influence the evaluation of one’s own body, in the sense of increasing the level of dissatisfaction. Research data supports the first hypothesis of our study, according to which social media pressure has a significant contribution to the process of internalizing the image of a thin body, and internalizing the image of a thin body guides the way in which the body image is built. And the pressure is even greater at this historical stage, when appearance is valued more at the expense of other capacities, skills, and Self-objectivation intensifies, especially among girls and women. More and more people consider it necessary to allocate time and resources to have a look that meets the criteria of the people they interact with, people in the online communities to which they belong, as evidenced by a study that investigated 1,983 adolescents (Mean age = 14.41 years), female and male, among Austrian, Belgian, Spanish, and South Korean, and that looked at aspects of media exposure in relation to body-image, moderated by self-objectivation (50). Because of social media pressure, especially for teenagers and young people, the natural process of social comparison is intensified. In order to define themselves, people evaluate their appearance in relation to the appearance of others (51). This mechanism has always had a role in evaluating one’s own body (52), people taking as a benchmark the images of other bodies to decide if they have a beautiful, attractive body, but nowadays, the standards associated with beautiful and attractive bodies are not only very high, they are unrealistic, impossible to achieve under natural conditions, with a healthy eating approach and dietary restrictions that do not pose a health risk. Women who resort to comparison in assessing body shape, considering more attractive appearances, are also those who are dissatisfied with their own bodies, are driven by the need to lose weight, and engage in restrictive eating behaviors, as observed in a study conducted on 321 women aged 19–25 years (53). The comparison itself, whether upward or downward, instills the need to have a slim body and the need to resort to diets. They are differentiated by the way the body is perceived; the first category of assessments is also associated with body dysfunction, negative attitudes, and thoughts related to appearance—issues not found in the second category. These aspects highlight that people who focus on attractive images on social media, considering them relevant, are more demanding with their own bodies. Also, the ability to manage negative emotions associated with comparison depends on both the quality of body image and eating behaviors. As reported in a study of 628 female college students, adaptive coping was associated with lower levels of body image deformity and behavioral disorders than maladaptive coping (54). Another study in this regard reports that social comparison, based on bodily aspects, produces much more intense effects than other comparison criteria, for example comparison based on intelligence (55). This highlights the role that aspects of appearance play in the process of building and reconstructing self-image, which are a defining facet. Given that more and more people are choosing social media platforms to spend their time (56), and considering that social media content often promotes images of thin bodies (57–59), as well as an intense concern for appearance, we can conclude that mass media is a powerful agent of influence. It significantly shapes the standards of appearance, attractiveness, and beauty, leading to a distorted perception of one’s own body (44, 60). These aspects, as well as those related above, allow us to formulate the second hypothesis of our study (H2).

*H2:* A high level of tendency to internalize certain standards of beauty and attractiveness of thin bodies is associated with a negative body image.

And this relationship impacts the quality of behavior, below we will highlight the effects it generates in terms of eating behavior. Self-denigrating attitudes lead to restrictive eating behaviors, a problem presented in other studies (61–63). According to DSM-5 (Diagnostic and Statistical Manual of Mental Disorders, Fifth Edition), we mention the most common forms of eating behavior disorder of this period: restrictive or avoidance eating behavior, anorexia nervosa, bulimia nervosa, excessive eating behavior (64). Restrictive eating behavior is the tendency to reduce the consumption of some foods or even eliminate others (65). Dietary restrictions are associated with cognitive factors, rather than physiological ones, associated with state of satiety or hunger (66). Thus, restrictive eating behavior is specific to people who are trying to control their physiological needs. Restrictive eating behavior is also found in people who are of normal weight, especially in women who appreciate that they have weight problems even when they do not have them (67, 68). The deformation of the perception of body shape leads to the deformation of the perception of weight, and which favors the installation of restrictive eating behavior. Over the past 30 years, there has been a lot of controversy surrounding the concept of diet or food restriction, highlighting both positive (weight loss makes greater contributions to diet risks) and negative (dietary restrictions favor food excesses; replacing diets with other techniques has led to inconsistent weight loss in people experiencing weight problems) (69, 70). In the current society, we are witnessing a concern regarding body image, guided by the need to correspond to landmarks of bodies’ attractiveness, but also by the repercussions of a consumer society, so we consider that the adoption of restrictive eating behaviors is indirectly encouraged. By social comparison, forming a negative self-image increases the tendency to adopt a diet, which is associated with the development of eating disorders (71, 72). In the study conducted by Soni et al. (73) on a population of 298 students, women and men, correlations between body image, self-esteem, media influence and attitudes towards food are highlighted. Young people who had inappropriate attitudes towards food were those who were more receptive to media influence, who had lower levels of self-esteem and a higher level of dissatisfaction with body image. The link between dietary restrictions, interest in diets and control over calories consumed and body image is present in both women and men, which highlights the role of society-imposed attractiveness benchmarks associated with slim bodies. Inappropriate attitudes towards food are a generating agent of young people’s tendency to form a distorted body opinion. Exposure to media related to weight loss has an impact on the correlation between body image and the presence of eating disorders (73). In the study conducted by Sanzari et al. (42) on a student population, the role of social media platforms (Snapchat, TikTok, and YouTube) at two points in time, 2015 and 2022, is analyzed, including aspects associated with the COVID-19 pandemic, on the relationship between the two psychological variables. The results point to more obvious disturbances in body image and eating behavior for 2022, due to the higher number of accounts held by young people. The study highlights the moderating role of the media content variable, the problems associated with body perception and those related to adopting an appropriate eating behavior being related to the quality of media materials, not to the time spent on these platforms, as initially expected. Simple exposure to media content that highlights the need to lose weight leads to the development of distorted body images, correlated with eating disorders, including the use of laxatives, invoking vomiting. To investigate how aspects of the relationship between body image and diet evolved, Ingolfsdottir et al. (74) conducted a study on Icelandic schoolchildren aged 16–19 years between 2000 and 2010. The study allowed the collection of data on 33,801 students, of both sexes, in order to draw a perspective on the psychological variables specific to this community, but also to capture the aspects related to overweight of Icelandic adolescents in relation to those of adolescents belonging to other cultural areas. The results indicated a higher level of overweight among Icelandic adolescents than Scandinavian adolescents, but lower than American adolescents. Negative body image is a strong predictor of the tendency to adopt a diet, both for women and men. The predisposition to acquire a diet is increasing among women and decreasing among men. In terms of body image, more women formed a negative perception compared to the male population. With age, the chances of following a diet increase, indicating that body image standards are likely to rise, leading to higher levels of dissatisfaction. Another conclusion of the study refers to the role that social media platforms have in terms of the landmarks they promote, so that messages that lead to the internalization of an ideal athletic body produce fewer negative effects on body image, consequently, reduce the possibility of adopting a diet, compared to those that lead to the internalization of an ideal slim body. Another study looking at body image, self-awareness and diet aspects was conducted on a population of 531 adolescents, aged 15–17, selected from Ankara schools. The internalization of an ideal slim body model, low levels of body appreciation, personal value are associated with dysfunctional body image and the presence of diets. We find lower scores in self-awareness, body image for dieting adolescents compared to those without dietary restrictions (75). The relationship between aspects of body image, self-esteem and eating behavior disorders is also highlighted in the population study of female students at Delhi University (76). The overall objective of the research was to identify predictors of eating behavior. Most of the participants showed dissatisfaction with their body shape (76.7%). Concern for body image and aspects related to social reflection, including how they were evaluated by family members, were the main factors generating eating disorders. A significant component of the predisposition to manifest dissatisfaction with physical aspects originates from the opinions that close people manifest, opinions that are internalized and generate inappropriate eating behaviors. Body perception is a predictor of eating behavior, people with a positive body image are less likely to have eating disorders. People with a positive body image have a high level of self-esteem, which contributes to well-being and quality of life. They also exhibit high self-acceptance, are less receptive to social media pressure to have a slim body, and adopt healthy lifestyles and appropriate eating behaviors (44). A population study of German adolescents, aged 11–17 years, presents data supporting the relationship between body shape distortions and the presence of restrictive behavior (77). Thus, adolescent girls who underestimated themselves in terms of appearance said they skipped meals, tried to control their weight through food, had dietary restrictions, manifested negative states after eating and states of annoyance associated with weight, the averages being much higher than those of adolescents who had a better perception of their own body. The study aimed at a comparative analysis of early-adolescents and adolescents, as well as gender differences. Early-adolescent girls showed a strong tendency to internalize an ideal image associated with slim bodies. Eating disorders, a tendency to deform body image, and negative moods associated with diet, body, and weight issues were much more prevalent in girls. Other studies also emphasize the association between body image, desire for a slim body, and eating behavior disorders (78, 79). The studies presented support the presence of a relationship between body shape perception and eating behavior disorders in different societies, including those with higher obesity rates, such as the USA, and those with lower incidence, like Japan (80). Attitudes and evaluations towards body shape, in association with the need to have a slim body, lead to the installation of appropriate eating behavior or problematic behavior, in both individuals with weight problems and those with a normal body mass index. Considering all the aspects presented above, we can formulate the third hypothesis of our study (H3).

*H3:* Negative perception of body shape is associated with a high intensity of restrictive eating behavior.

Considering the role of external social factors in the process of assessing body shape, as well as the association between it and restrictive eating behavior, we illustrate associated aspects of internalization of body imagery considered to be attractive in the social media space. Different scholars have studied the concept of body image over years in multiple contexts, highlighting positive or negative perceptions over it. The body appreciation has been considered one of the key elements capable to operationalize the positive body image, being defined as the ability to form a set of positive attitudes towards one’s own body, which is associated with its appreciation and acceptance, without validating the beauty models present on social media (13, 81). In a general view, a positive body image involves respecting, honoring, loving, and displaying gratitude towards the features, functionality, and health of the body (82). According to different studies, there is a positive connection between body appreciation and different other constructs like: favorable appearance evaluation, self-esteem, optimism, proactive coping, positive affect, life satisfaction, and self-compassion (81, 83–86). In the same time, body appreciation is inversely related to body dissatisfaction, social physique anxiety, body image avoidance, body shame, body surveillance, body checking behaviors, and internalization of societal appearance ideals (87–92). In addition, an inversed relationship was found between body appreciation and different pathologies like eating disorder symptomatology, neuroticism and maladaptive perfectionism (90, 93, 94). Body appreciation as it is measured with the help of Body Appreciation Scale (BAS) does not rely simply on the absence of negative body image or the experience of self-perceived attractiveness, but a kind of valuation of individuals body image and manifestation of criticism about unrealistic body images promoted by the media (95, 96). Actually, within the scientific literature there are many studies that are demonstrating the link between internalization of thin body or other cultural models promoted over the media and body appreciation. Thus, body appreciation is involved in the development of the ability to deconstruct unrealistic media images (97, 98). In a study made in 2013, Halliwell observed that the protective effect of high body appreciation is extended to women known to be vulnerable to media exposure-those who have internalized the thin ideal (99). Specifically, women subjects, that have endorsed the thin ideal and had low body appreciation after they have been exposed to thin female model images, reported larger appearance discrepancies and placed more importance on their appearance discrepancies. In the same time, women that have endorsed the thin ideal but had high levels of body appreciation do not give the same importance to their appearance discrepancies. On another line of research, a study made on a sample of 228 black college women from USA has shown that levels of higher body appreciation were linked to less history of weight related teasing, lower eating, weight and shape concerns, and lower Western beauty ideal internalization (100). So, it become clear, that the link between body appreciation and internalization of different cultural predefined standards relating with thin bodies, fit appearance etc. goes in both ways – higher internalization means lower body appreciation, higher body appreciation means lower internalization. The results of research conducted by Bordo in 2003, show that individuals who internalize the perception that excess body weight and the appearance of a fat body are linked with lower morality, lack of willpower and control, and personal inadequacy find it difficult to accept, love, and respect their bodies (101, 102).

So, again, it seems that higher internalization of such perception about “must have thin body” standards is connected with lower levels of body appreciation. Other studies like the research conducted by Alleva, Veldhuis, and Martijn in 2016, on a sample of Dutch female respondents with ages between 18 and 28 years, are showing that women that are focusing on their body functionality and as a consequence manifesting body appreciation have been able to buffer any potentially negative effects of media exposure (103). Finally, a study made on 266 women respondents from Australia showed that greater perceived body acceptance by others and self-compassion, and lower appearance media consumption, self-objectification, social comparison, and thin-ideal internalization were related to greater body appreciation (104). Another study conducted by the same authors showed that body appreciation predicted less change in body dissatisfaction following exposure. Participants with low body appreciation experienced increased body dissatisfaction, while those with high body appreciation did not (105). Taking account of all of the above, we can issue the fourth hypothesis of our study (H4).

*H4:* A high level of the tendency to internalize standards of beauty and attractiveness of thin bodies is associated with a lower level of Body Appreciation.

The tendency of depreciation of body image associated with restrictive eating disorders, aspects supported by previous studies, which we have reported in the previous sections, leads us to turn our attention to aspects related to emotional eating disorder. The complexity of the concept of emotional eating disorder is given by the combination of perspectives from which it can be explained and described, meaning social psychology, clinical psychology, psychotherapy, medical psychology and nutrition (106–109). We can define emotional eating behavior as “as eating in response to negative emotions” or as the tendency to overeat due to the inability to manage emotions (110, p. 290). Food is not only consumed to satisfy physiological needs, but also to respond to emotional, psychological needs, aspects related in research aimed at investigating psychological aspects in relation to obesity. Of the 256 obese patients evaluated, 49% had depression and 56% had anxiety, which explains the role of psychological comorbidities in explaining eating behavior (111). The relationships between negative emotions, anxiety, depression, and the presence of emotional eating behavior led to the creation of the Emotional Eating Scale (EES) by Arnow et al. (112), which includes three subscales: Anger/Frustration, Anxiety, and Depression. The Dutch Eating Behavior Questionnaire (DEBQ) allows the examination of aspects related to eating disorders, presenting information on three categories: restrained, emotional, and external eating (113). The onset of emotional eating behavior is based on both negative and positive emotions support both variants (114–116). Binge eating is more commonly determined by negative emotions compared to positive ones (117). Binge eating disorders involve abnormally high food consumption in a very short time and is associated with psychological and non-psychological factors (118). Research data supports the idea that binge eating is an unhealthy way to compensate for negative emotions and promote emotional behavior disorder (119). The data presented above point to the link between emotions and the quality of eating behavior, aspects that are also related to the quality of body appreciation (BA), a variable described in the section dedicated to the fourth hypothesis of our study. Thus, in a recent study, 301 participants, women and men, without declared pathologies, physical (chronic conditions) or mental (including eating disorders), were evaluated in terms of body appreciation, eating behaviors, depression, anxiety, and stress. The results indicate that high anxiety, depression, and stress (distress) scores and low body appreciation scores were associated with eating behavior disorders (110). Women had higher scores on levels of anxiety, stress, and a tendency to develop eating disorders, and lower levels of body appreciation than men. A longitudinal study, within the University of North Carolina Greensboro, NC, United States, started from the investigation of 445 participants, selected from childcare centers and care centers for mothers with children, analyzing aspects related to emotional eating, emotion regulation and negative body image (120). Measurement of psychological variables began at age 2 and continued into adolescence. The analysis of 138 adolescents indicated that there were no significant differences in eating behavior, depending on weight, and the presence of differences in body appreciation, depending on gender, with girls showing lower levels of body appreciation. Differences in body appreciation were also observed, depending on weight. In the case of adolescents who negatively appreciated their body, the regulation of emotions led to a decrease in the tendency to adopt emotional eating behavior. Thus, we highlight the role that body appreciation has in relation to the tendency to adopt emotional behaviors from the first years of life, tendencies that are preserved in adolescence, and later in adulthood, and that have an impact on public health. As observed in the study conducted by Bucchianeri (121), problems with appearance are predictors of mental health and eating behaviors. Another study highlighting the impact that problems associated with emotional eating and those associated with body appreciation, in relation to attachment type, have on the manner in which quality of life is assessed, is the one conducted by Laporta-Herrero et al. (122). Data collected from 260 adolescents, including 129 participants without clinical problems and 131 participants with eating disorders, receiving treatment in a specialized center in Spain. Secure attachment is associated with a positive appreciation for both categories of adolescents, and a good body appreciation correlates with body image quality of life. Adolescents with eating disorders showed a better quality of life relative to body image when they had favorable relationships with fathers, the same bound was observed for adolescents without eating problems, only in relation to mothers. In order to form an adequate body image, as well as healthy attitudes towards it, adolescents need a quality relationship with their parents, based on trust, which will propagate on how they perceive and evaluate their life. Quality interpersonal relationships are based on people’s ability to provide social and emotional support, so people who benefit from compassion and/or practice self-compassion will evaluate themselves by using positive terms (123), and will be less tempted to develop eating disorders (124). The compassion that mothers showed in relation to their daughters led to a good appreciation of the body, as well as lower chances of adopting emotional eating (125). The data of the studies presented highlight the role of social factors in building body appreciation, as well as managing emotions related to eating behavior. Thus, strategies aimed at reducing the frequency of eating behavior disorders have as a starting point the improvement of the way in which the body is perceived. In this regard, a study was conducted on a population of obese women (mean age = 41.4 years) who received treatment for obesity. The results indicated that managing aspects of body satisfaction to improve it in those who manifested the highest scores in the emotional eating variable led to better results compared to obese women who exhibited normal levels of emotional eating (126). The aspects presented above allow us to formulate the fifth hypothesis of our study (H5).

*H5:* A lower level of Body Appreciation is associated with a high level of intensity of Emotional Eating Behavior.

Studies conducted in Europe (74, 77, 103), Asia (76, 80, 127, 128), and US (100, 120) report the trend of increasing numbers of people, from increasingly younger ages, who develop distorted body image and low levels of body appreciation, as well as eating disorders, as presented in the sections above of this paper. The data is worrying given the impact that the quality of body appreciation has on the quality of mental health (129). The perception and evaluation of the body being strongly correlated with physical health and the quality of behaviors (130, 131). If in the past, problems related to body shape were rather associated with adolescence, being related to physiological and hormonal changes, nowadays we witness a generalization of these disorders, being encountered including in mature adults, and the causes are, this time, external—the need to face standards promoted on social media or family pressure, friends (27). Although we do not identify the same causes, more and more people, from children to adults, define themselves in negative terms, devalue and denigrate themselves (95, 96, 120, 122). A population study of women highlights differences in this regard. Women aged 25–68 showed concern for weight control and a higher level of inappropriate eating behaviors, even in the absence of issues associated with body shame, compared to those aged 18–24, whose concern for weight was associated with high levels of body shame and the presence of disordered eating behaviors (132). The high level of body shame indicates the presence of negative attitudes towards one’s own body, a negative evaluation of body shape, lack of body appreciation. As evidenced by studies reporting the association between high levels of body shame, low levels of body appreciation and the presence of bulimia nervosa (131, 133, 134). The presence of bodily shame will negatively influence the level of mental health, expressed by the valence of attitudes towards oneself, the manner of reporting towards physical appearance, the quality of eating habits and actions (135). Another explanation for the intensification of body dissatisfactions, which are also associated with body shame, and body surveillance, is self-objectification, a bound, validated by data reported in the study of 371 people, licensed in Psychology from University of Turin (136). Self-objectification is specific to people who use the term object when they relate to themselves, instead of subject, an object to be observed and analyzed by others (137), an object whose existence depends on the pleasure of others (138). Thus, there is a division of intrinsic human value from corporeal value, the emphasis being placed on the second component, an intensification of self-objectification being associated with a high level of need for body supervision and a low level of body appreciation (139). Self-objectification originates from the women sexual objectivation, a perspective that presents the woman only as a sexual object or associated with social functions, cancelling out the aspects of personality that give her specificity (140). In the absence of self-objectification, we notice a lower tendency of people to evaluate their body, taking into account the opinions about their own appearance, with negative content, that are expressed by others. Data from a qualitative study indicate that adolescents, girls and boys, who have a positive self-image, did not consider messages with negative connotations (97). A good body appreciation is a healthy way to cope with external factors, such as media exposure, to diminish the possibility of developing body dissatisfactions (85, 99). In terms of gender differentiation, men exhibit higher levels of body appreciation (105, 141), but with age, women begin to value their bodies more, compared to men, whose assessment remains constant with age (85). In the light of all of the above we can issue our sixth hypothesis of our study (H6).

*H6:* A lower level of Body Shape Appreciation is associated with a lower level of Body Appreciation.

**Figure 1:**
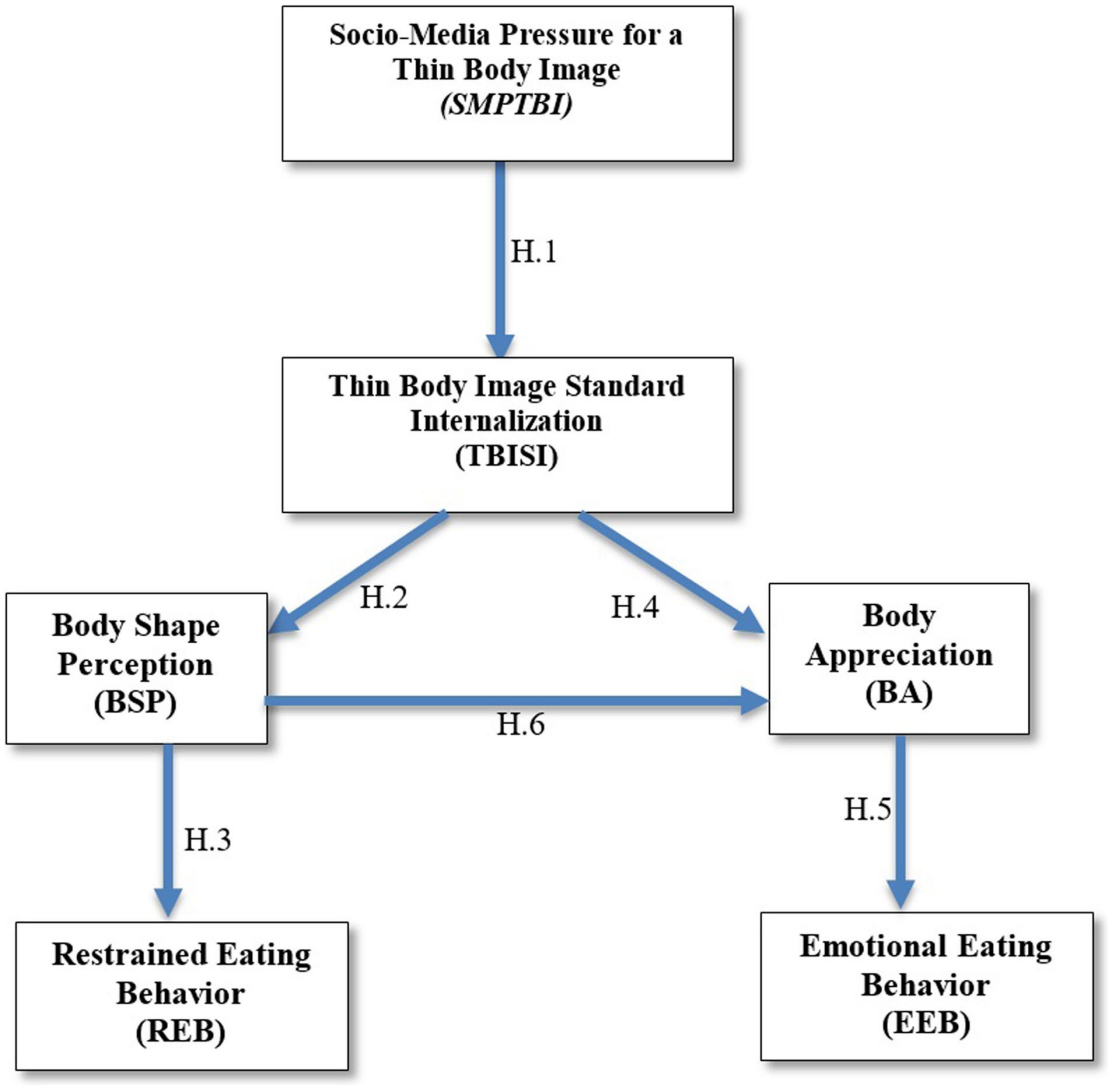
Theoretical model proposed, highlighting the relationship between identified variables.

The presentation of the relationships between psychological variables, consequently the hypotheses underlying our study can be found in Figure 1 in the form of a theoretical proposed model.

## 3 Research design and methodology

### 3.1 Research design

In the following section we will present the main aspects regarding the research design and methodology used. The present study has as main characteristic the fact that allows the research of relationships. Within the research we may highlight the following psychological variables: Socio-Media Pressure for a Thin Body Image (SMPTB), Body Appreciation (BA), Body Shape Perception (BSP), Restrained Eating Behavior (REB), and Emotional Eating Behavior (EEB). The demographic variables taken into consideration were: age and gender. From this point of view, we have to highlight the fact that we have constructed our sample starting from a statistical population made of students coming from a number of four higher education institutions.

The sampling method used in the case of present research is convenience sampling. This method involves selecting participants based on their accessibility and willingness to respond. The questionnaire was distributed online, making it accessible to students from the four universities. Only students from the specified universities were targeted. Participation was voluntary, meaning only those who were interested and available completed the questionnaire.

The final sample has 38.8% male respondents and 61.2% female respondents, with ages between 18 and 21 years (52% of the respondents), 22–25 years (22% of the respondents) and 26–50 years (26% of the respondents). The differences between the number of female and male respondents is due to the fact that the majority of students came from social sciences and humanities specializations, and the higher proportion of women within the student population in Romania (according to Romanian Ministry of Education reports, in the year 2020/2021 there has been 43.3% female students in comparison with only 33.9% male students – numbers calculated by relating the number of students in the country (excluding foreign students) to the population in the 19–23 age group) (142). The establishment of association relationships was based on specialized literature relevant to the investigated topic, as it can be seen within the theoretical model proposed.

### 3.2 Measuring instruments

To measure Socio-Media Pressure for a Thin Body Image (SMPTB) and Thin Body Image Standard Internalization (TBISI) we applied the Sociocultural Attitudes Towards Appearance Questionnaire-4 (SATAQ-4) scale (27). The questionnaire includes the following subscales: Internalization: Thin/Low Body Fat, Internalization: Muscular, Internalization: General Attractiveness, Pressures: Family, Pressures: Media, Pressures: Peers, and Pressures: Significant Others. To assess the social media pressure, young people feel regarding beauty and attractiveness standards, we used the Pressures: Media subscale, and to assess the tendency to internalize beauty and attractiveness standards of slim bodies, we used Internalization: Thin/Low Body Fat subscales. The scale shows five response steps as follows: Definitely Disagree = 1; Mostly Disagree = 2; Neither Agree No Disagree = 3; Mostly Agree = 4; Definitely Agree = 5. The assessment of the Body Shape Perception (BSP) variable was performed using the Body Shape Questionnaire. The scale contains 16 items aimed at self-assessing aspects associated with body shape in the last 4 weeks (143). The participants opted for one of the answer options with step: 1 = never, up to 6 = always. A high score is associated with a tendency to worry and dissatisfaction with body shape. We used the Body Appreciation Scale-2, developed by Tylka and Wood-Barcalow (13), to measure body appreciation (BA). The scale comprises 10 items and presents five response steps, where the value 1 = never and 5 = always. A high score indicates a high level of body appreciation. The variables Restrained Eating Behavior (REB) and Emotional Eating Behavior (EEB) were measured using The Dutch Eating Behavior Questionnaire (DEBQ) for Assessment of Restrained, Emotional, and External Eating Behavior (113). The questionnaire includes scales for restrained, emotional, and external eating. We only used items associated with restrictive and emotional behavior. The answer was Likert, from 1 = never, to 5 = very often. High scores indicate the presence of restrictive or emotional behavior. Annex number one presents the variables, items and their corresponding sources.

### 3.3 Procedure

Data were collected using an online survey form (Google Forms) between January and April 2024. The students were assured of confidentiality in accordance with the provisions in force of the Regulation on the protection of individuals with regard to the processing of personal data and on the free movement of such data. Clicking onto the questionnaire indicated consent to participate.

## 4 Results

The collected data was processed using IBM SPSS Statistics version 29.0. The program allowed both descriptive data analysis (testing the internal consistency of all psychological variables and distribution) and inferential analysis (correlation analysis and comparative analysis by gender of respondents of *F* and *t* Tests for Independent Samples). In the first phase, the internal consistency of psychological variables was analyzed. In Table 1, information for each variable is provided, with Cronbach’s Alpha coefficient values being relatively high. This indicates good to excellent internal consistency for the analyzed variables, demonstrating that the measurement scales have suitable internal consistency for use in data analysis.

**Table 1:** Cronbach’s Alpha coefficient values and descriptive statistics for the analyzed variables.

Variables	Cronbach’s Alpha	Mean	Minim and maxim possible variables values	Std. Dev.	Skewness	Kurtosis
Socio-Media Pressure for a Thin Body Image (SMPTB)	0.910	16.666	4–20	4.460	−0.130	−1.242
Thin Body Image Standard Internalization (TBISI)	0.832	20.581	5–25	5.223	−0.286	−0.821
Body Appreciation (BA)	0.966	44.358	10–50	13.012	0.566	−0.999
Body Shape Perception (BSP)	0.960	36.915	16–96	9.496	−0.176	−1.118
Restrained Eating Behavior (REB)	0.954	26.259	10–50	7.120	0.279	−0.954
Emotional Eating Behavior (EEB)	0.974	42.795	13–65	11.108	0.410	−1.032

**Figure 2:**
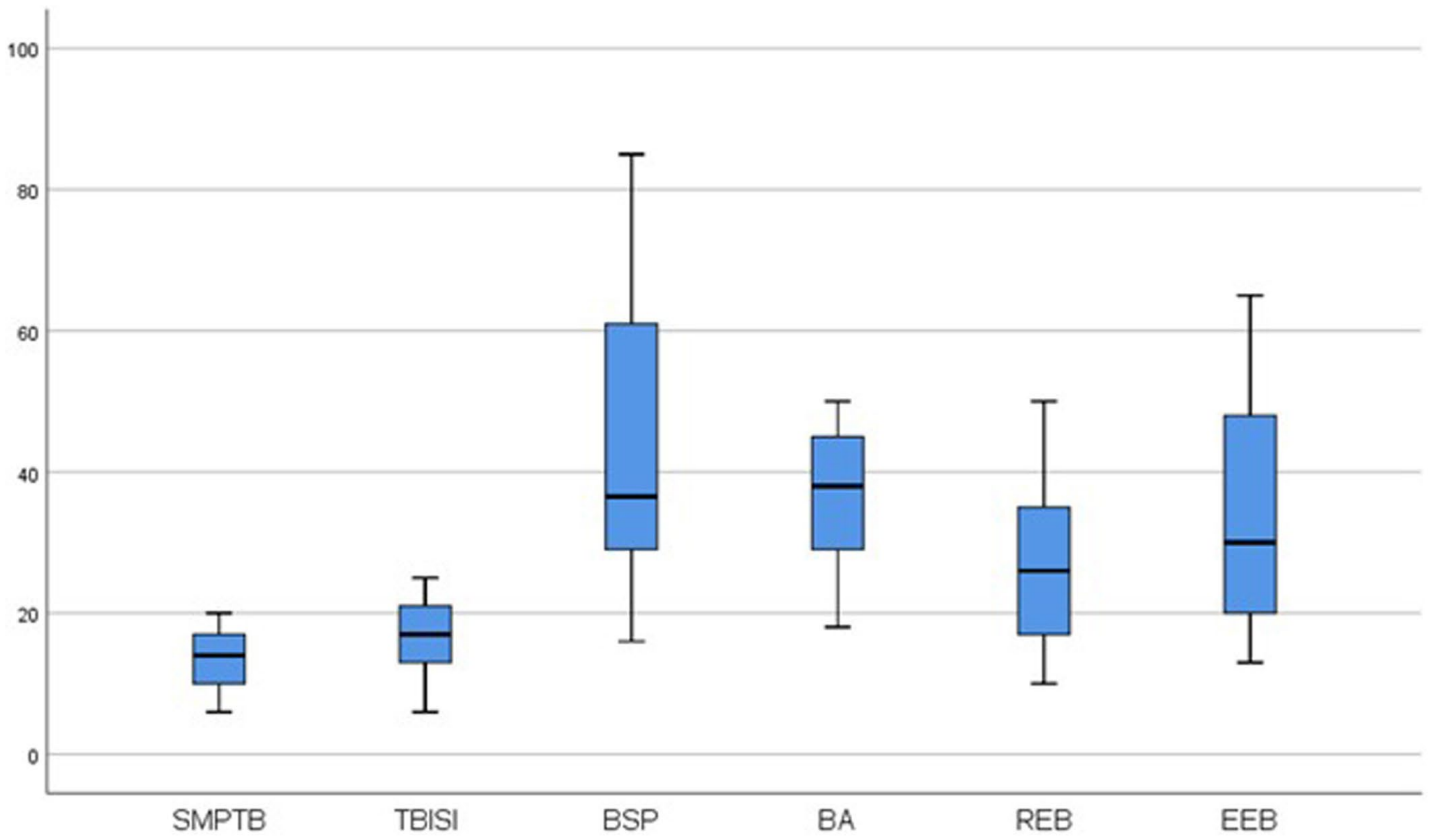
Box plot graphs related to the analyzed variables. SMPTB, socio-media pressure for a thin body image; TBISI, thin body image standard internalization; BSP, body shape perception; BA, body appreciation; REB, restrained eating behavior; EEB, emotional eating behavior.

To assess the shape of the distribution we used the asymmetry coefficient and the vaulting or flattening coefficient—Skewness and Kurtosis (144). Skewness acceptable range for values is [−2, +2] and Kurtosis acceptable range for values is [−3, +3] (145, 146). Thus, the values of the two indicators in Table 1 indicates that the data is normally distributed. It can be found that in the case of the analyzed variables there are no extreme values that distort the mean (Figure 2).

For variable testing, the Pearson correlation analysis was used, which allowed testing the intensity of the connection between psychological variables (Figure 2).

**Figure 3:**
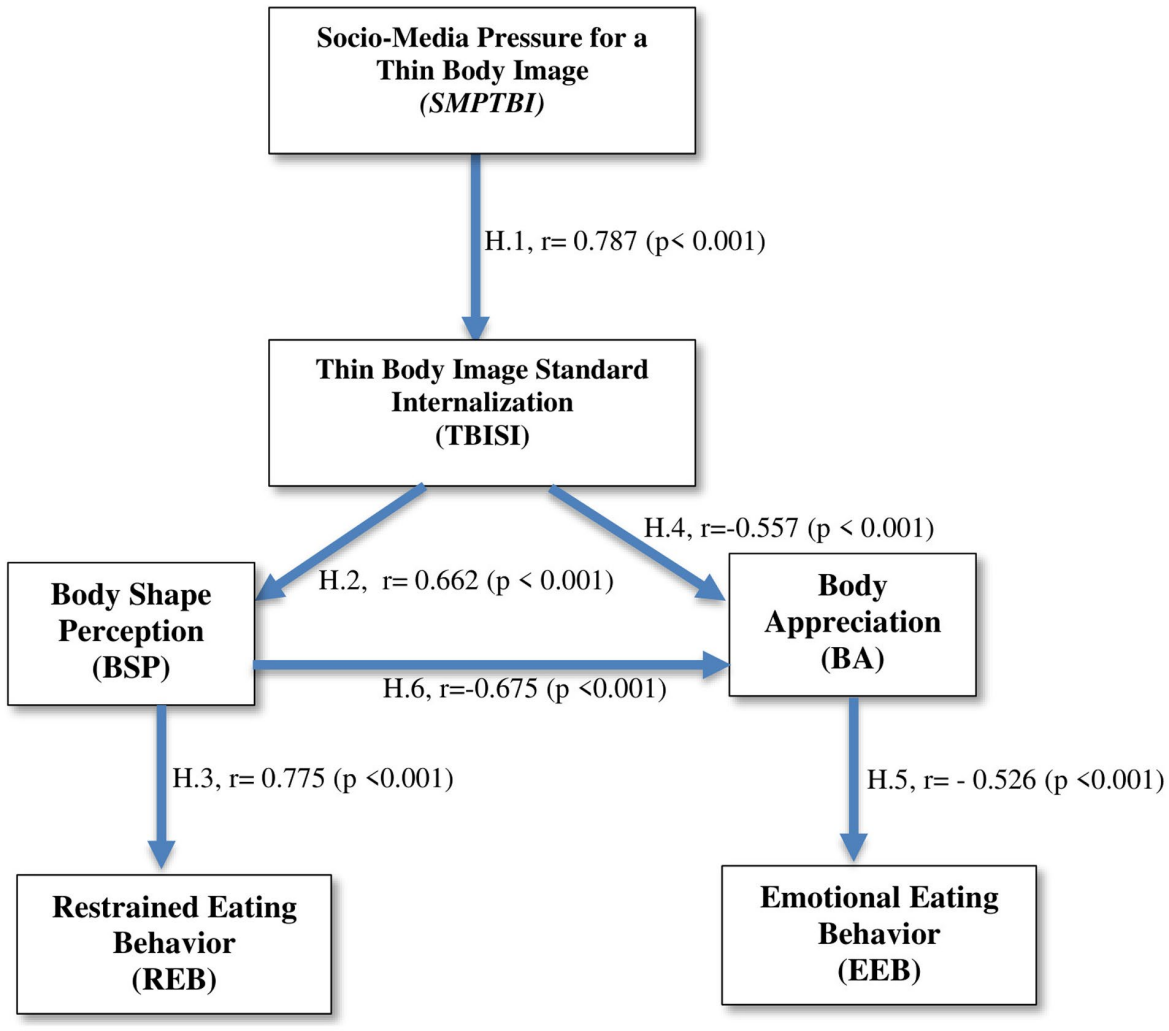
The empirical model validated by the relationship between considered variables.

In the light of the obtained results we can present the theoretical model proposed, validated by the values for each relationship between the concerned variables, as it can be seen in Figure 3.

In the following we will present the results corresponding to each advanced hypothesis, in order to assess their validation. Thus, hypothesis H.1 stated that there is a direct, positive and strong relationship, statistically significant between the social media pressure young people feel about ideal body image and the tendency to internalize standards of beauty and attractiveness of slim bodies (*r* = 0.787, *p* < 0.001). The materiality value allows us to validate the research hypothesis. A high level of pressure that young people feel about the ideal of body image leads to the internalization of this standard of beauty. Thus, exposure to images and videos of thin and attractive bodies is perceived as a compulsion to follow those benchmarks, a unique standard of beauty. Social media content dictates the process of forming an ideal body image. Targeting consists of indicating aspects related to appearance that need to be relevant, meaningful, showing the way forward to have a beautiful and attractive body. People who attach considerable importance to these messages will be more vulnerable to assimilate passively, without subjecting them to evaluation, critical thinking and without relating them to the particularities of their own body. The second hypothesis stated that a high level of tendency to internalize certain standards of beauty and attractiveness of thin bodies is associated with a negative body image. From this point of view our results helps us to identify a direct, positive, medium intensity, statistically significant relationship between the tendency to internalize standards of beauty and attractiveness of thin bodies and the negative perception of body shape (*r* = 0.662, *p* < 0.001). The hypothesis is confirmed, which is confirmed by the materiality value. A high level of internalization of beauty and attractiveness standards promoted in social media causes a negative perception of body shape. The approach of internalizing a model of beauty and attractiveness changes the personal indicators of body analysis, thus creating new evaluation criteria, much more demanding, high demands appear in relation to one’s own body. The comparison process is carried out between the image created by social media, which is embellished to match marketing strategies and the real body image, which does not benefit from improvement programs or filters. This leads, in most cases, to the formation of a negative perception of body shape. The third hypothesis stated that negative perception of body shape is associated with a high intensity of restrictive eating behavior. We found that there is a direct, positive, strong, and statistically significant relationship between negative body shape perception and intensity of restrictive eating behavior (*r* = 0.755, *p* < 0.001). The materiality value allows the initial assumption to be confirmed. A high level of negative perception of body shape leads to dietary restrictions or avoidance of certain categories of food. Reducing the level of dissatisfaction with one’s own body is achieved by trying to control body weight. The eating disorder is installed as a repercussion of the need to restore an inner balance, by modifying aspects related to appearance. The trend is even more obvious in today’s society, given the price placed on the manner in which a person looks, the manner in which the body is displayed illustrates a marketing strategy in itself, of promoting oneself. And, while in the past these tendencies were found among public figures or those associated with political, artistic, or beauty fields, today it is common and considerably more concerning, as personal value is increasingly associated with the ability to meet certain beauty standards, even at younger ages. The fourth hypothesis stated that a lower level of Body Appreciation is associated with a high level of intensity of Emotional Eating Behavior. We identify an inversely proportional relationship, of medium intensity, but statistically significant between the two variables (*r* = −0.557, *p* < 0.001). The hypothesis is confirmed, given the value of p. A high level of internalization of a standard of beauty and attractiveness associated with a slim body leads to negative body appraisals. The assimilation of an unrealistic model, given the fact that images and videos on social media promote thin bodies, worked at the gym, and in reality, there are a variety of types of silhouettes, favors the appearance of negative attitudes and thoughts in relation to one’s own body. The inability to match a standard translates into self-discredit, contempt for one’s appearance. The large discrepancy between the ideal image and the real image creates discomfort, tension that reflects the valence of body evaluation. The fifth hypothesis stated that a lower level of body appreciation is associated with a high level of intensity of emotional eating behavior. We identify an inversely proportional relationship, of medium intensity, statistically significant, between body appreciation and intensity of emotional eating behavior (*r* = −0.526, *p* < 0.001). The materiality value indicates that the hypothesis is confirmed. A low level of body appreciation leads to the onset of emotional eating behavior. Negative emotional states associated with low body esteem are removed with the help of food. Eating is the way to lessen negative emotions. Food is a handy option, an easily available resource, which is consumed in order to compensate for the tension felt, anxiety, anger or anger in relation to the body. A positive body image means accepting and respecting the body, regardless of the changes it is going through, with less chance of changing perceptions and attitudes in relation to external factors. The sixth hypothesis stated that a lower level of body shape appreciation is associated with a lower level of body appreciation. From this respect, we found that there is an inverse proportional relationship of medium intensity between Body Shape Perception and Body Appreciation (*r* = −0.675, *p* < 0.001). A high level on the BSP scale indicates dissatisfaction with one’s own body, and a high BA score is associated with the ability to judge one’s appearance. Thus, a high level of bodily appreciation is associated with appropriate perceptions related to physical aspects. Materiality validation allows validation of the initial hypothesis. Negative assessment of body shape is associated with self-devaluation attitudes in relation to the body. People who do not have a good opinion of their own body also develop inappropriate attitudes towards it, of disgust and discredit. A positive body image means accepting and respecting the body, regardless of the changes it is going through, with less chance of changing perceptions and attitudes in relation to external factors (Table 2).

**Table 2:** Comparative analysis of psychological variables by respondent gender using independent samples *t*-test and Levene’s test.

Independent samples test									
	Levene’s test for equality of variances		*t*-test for equality of means						
	*F*	*p* (Sig.)	*t*	df	*p* (Sig. 2-tailed)	Mean difference	Std. error difference	95% confidence interval of the difference	
								Lower	Upper
Socio-Media Pressure for a Thin Body Image (SMPTB)	4.733	0.030	−3.239	612	0.001	−1.27726	0.39431	−2.05163	−0.50290
Thin Body Image Standard Internalization (TBISI)	28.020	0.000	−2.534	612	0.012	−1.17415	0.46332	−2.08403	−0.26426
Body Shape Perception (BSP)	81.655	0.000	−7.540	612	0.000	−12.87091	1.70694	−16.22308	−9.51873
Body Appreciation (BA)	19.416	0.000	3.668	612	0.000	3.07195	0.83760	1.42704	4.71686
Restrained Eating Behavior (REB)	13.454	0.000	−5.339	612	0.000	−5.17430	0.96920	−7.07766	−3.27094
Emotional Eating Behavior (EEB)	32.783	0.000	−7.068	612	0.000	−9.76124	1.38109	−12.47348	−7.04899

It can be found that there are statistically significant differences between women and men in all variables in the model, with women having a higher average for all variables analyzed except the score for BA where men have a higher average than women. Statistically significant differences in body appreciation between women and men are also reported in previous studies (147, 148). Women averaged SMPTB, TBISI, BSP, REB and EEB given the higher share of materials exhibited in the social media space for them, on the one hand, and the female population is more interested in topics associated with body shape, diets, areas of activity associated with beauty (149–151), compared to the male population. Thus, the pressure that women feel to have a body that meets social media standards is greater than the pressure that men feel (121, 152), consequently the internalization of beauty standards associated with thin bodies is greater, which favors the development of body dissatisfaction and food restrictions or emotional eating. The strong tendency of women to internalize an image of slim bodies also occurs in societies where there are no weight problems, an aspect reported in a population study of women in Japan (153). Another aspect that highlights gender differences in internalizing a standard of beauty and attractiveness refers to the fact that women are more concerned about having a slim body, adhere to models of appearance like this, while men are concerned about body muscle (78). The content of the materials promoted in social media being customized in this sense, we find several images and videos with women whose bodies are thin and with men whose bodies are worked out at the gym.

## 5 Discussion

The images exposed in social media become benchmarks in terms of the standard of attractiveness and beauty, viewing images and videos showing thin bodies intensifies the comparison process, becoming more interested in the shape of their own body and manifesting a high degree of internalization of beauty standards (154–158). The pressure of social media is all the stronger because of the increasing role of social media platforms in people’s lives, and because of unrealistic standards related to the appearance and promotion through them. Viewing photos or videos showing attractive bodies creates discomfort among both men and women. The discrepancy between the images in social media and the real image is large and frustration sets in, given that the images are generally valid, are not customized, do not take into account all physical typologies. Aspects that we also find in the study conducted by Pritchard and Button (159), whose results indicate that both women and men expressed feelings of dissatisfaction with weight, when they were exposed to images related to the ideal of appearance, and these were more intense than when they looked at body-positive imagery. Genetically, each person has a minimum of weight that they can reach, so there are greater or lesser chances of corresponding to a standard of beauty, but which cannot be fully controlled. In a study conducted on the population of women, it is observed that they cannot get to have an appearance associated with the ideal image, which implies a minimum in terms of weight, and at the same time maintain their health (160). There are data that confirm our perspective, the need to present images that support diversity, multiple forms of beauty. Some studies (161, 162) show that exposure to positive body images is associated with high levels of body satisfaction in both women and men. One of the explanations why we are witnessing a strong tendency, especially of adolescents and young people, to assimilate unrealistic standards of beauty and attractiveness, are movements such as thinspiration, or fitspiration. Although they aim to motivate followers to get a body similar to images on social media, they only increase the level of dissatisfaction with their own body (163–165). The intense tendency to internalize a standard of attractiveness and beauty associated with thin bodies is associated with a negative evaluation of body shape. The desire to have a slim body, the ideal facet of body image, compared to the real facet of body shape perception leads to increased levels of dissatisfaction. Especially in contemporary society, when more emphasis is placed on external facets at the expense of inner resources. Pictures uploaded to social networks are edited beforehand, which is explained by the internalization of beauty stereotypes and the need to conform to high standards (166). Internalizing an ideal of the slim body implies from the start an excessive preoccupation with body shape, weight (167), but also a greater attention paid to the manner in which the body is portrayed in the social-media space (168). And the exaggerated preoccupation with body shape is associated with the creation of a body ideal by constantly referring to the standards related to appearance in the social media space, which leads to body dissatisfaction The interest in displaying a body image that corresponds to the ideal of beauty and attractiveness of this historical stage is materialized in the act of processing and editing the photos that are displayed in the social media space (169, 170). A consistent component of self-presentation refers to the impression that young people create in the social media space, by selecting and posting photos that correspond to cultural and historical standards. In recent studies, young people worried about their body shape will edit the pictures they upload on social media platforms (171, 172), to display the ideal body facet, the one they want, the facet that is appreciated and validated by their community. Editing programs and filters are used to mask imperfections in appearance (163), in the case of those who are dissatisfied with body shape, and exposure to these edited photos leads to body dissatisfaction. A study of 144 teenage girls reports that simply examining edited photos posted on social media leads to a negative assessment of body shape (173). Students are more vulnerable to the need to make a good impression through the profile created on social media platforms. One possible explanation is that appearance is associated with popularity, young people who receive positive reviews for uploaded photos have a higher status in their community and better chances to integrate socially. Another explanation starts from the social representation of femininity, which is associated with the need to have a lean body, and the social representation of masculinity, which indicates the need to have a fit body (174, 175). The internalization of an ideal of the slim and fit body is associated with high standards of appearance, but also with harsh criteria of comparison. Studies indicate an increased level of body dissatisfaction based on comparison with famous people in the media, popular on social networks, especially those associated with the field of beauty (44, 154, 176). The negative evaluation of body shape favors restrictive eating behaviors. People dissatisfied with physical aspects resort to dietary restrictions, prohibitions or diminishing the consumption of prohibited foods in order to improve their body shape, to reduce the discrepancy between the way they perceive themselves and the way they would like to look. Negative body shape perception is a strong predictor of dietary restrictions (76), for both women and men, the chances of dieting increase with age, which is explained by rising beauty standards (74). The approach of assessing body shape is doubled by that of forming attitudes in this sense. Thus, positive attitudes occur in relation to the image of thin bodies and negative attitudes in relation to fat bodies (177). And the valence of attitudes towards body shape directs the valence of attitudes towards food. Thoughts and beliefs about one’s own body lead people to appreciate what categories of foods can be eaten, because they allow maintaining a body shape, and those that need to be eliminated because they prevent them from reaching the desired body shape. As a result, negative attitudes towards high-calorie foods and positive attitudes towards those with a low caloric index develop (178). Body shape is evaluated according to how close or far it moves away from the desired image, and this assessment influences attitudes towards food, which are divided into two categories, those allowed and those prohibited. The deformation of body shape accentuates the tendency to restrict eating behavior (66, 131, 153, 179). The strong internalization of certain standards of beauty and attractiveness of slim bodies is associated with a negative appreciation of the body. The stronger the desire to have thin bodies as a model, the stronger the tendency to depreciate one’s own body. The approval of beauty models associated with thin bodies promoted on social media increases vulnerability when exposed to these images, as well as the degree of deformation of aspects (99). Creating an ideal of beauty that does not correspond to the personal physiological structure will lead to unrealistic body appraisals. Studies indicate that social beauty standards influence one’s personal ideal of beauty, impacting one’s level of self-esteem (180). Exposure to images and videos with attractive, beautiful bodies, usually of public figures, models or artists, causes the onset of negative emotions in relation to one’s own appearance, shame, guilt, disgust. Comparing one’s own body with the one in the images that appear in the social media space creates distress, especially since the standards are unrealistic, the materials are processed, the photos are filtered, they are edited. In this regard, a study conducted by Harvey et al. (181) highlight an increased sensitivity to disgust towards one’s own body and food in people with eating disorders. Thus, it is observed that the improvement of problems associated with eating disorders is associated with lower score of disgust towards one’s own body (181). The relationship between the presence of disgust and the adoption of dysfunctional eating behaviors can also be found in the more recent study (182), which presents data on 2,317 Italian participants, 57% of whom are women, aged 17–69 years. The data suggests that among the non-obese population, restrictive eating behaviors occur in those with a high body mass index, as a result of a high level of sensitivity to disgust, which is associated with a high level of self-disgust. When people overestimate their weight, especially women, starting from the model of a slim body image, there is also a tendency to increase the level of disgust with appearance, as well as the need to find a quick solution to improve appearance. As evidenced by the study conducted by Anna Brytek-Matera et al. (183), people who had eating disorders were also those who had developed a dysfunctional image of what the ideal body illustrates, as well as a distorted perception of the body. In the same direction, another negative emotion expressed in relation to one’s own body, shame, is associated with the presence of eating disorders, the anticipation of body shame representing a deficient way to diminish the chances of eating problems (184). Thus, we can specify that reducing the time spent on social media platforms can have contributions in terms of attitudes and emotions that are conceived in relation to appearance, with beneficial effects on food decisions and behaviors. Respect for one’s own body leads to positive attitudes towards food, consequently also to adequate, healthy eating behaviors, a low level of body appreciation is associated with eating behavior disorders, body image deformity, the presence of anxiety and depression (81). Eating behavior disorders are specific to both adolescents and young adults (120, 185). The data is worrying, given that problems associated with eating behavior in childhood persist into adolescence and then continue into adulthood (186). At this historic stage an increasing number of children and adolescents are experiencing mental health problems (187–190) anxiety, depression, suicide attempts, eating disorders, obesity, alcohol addictions and prohibited substances. A possible explanation for this phenomenon is given by the pressure placed on the younger generation, the level of demands being higher and higher, which can be associated with perfectionism, the need to be perfect, to act perfectly (191). Another explanation is offered by exposure to social media content from a very young age, which on the one hand influences their benchmarks, including those related to appearance, guides their behaviors and decisions. Given the fact that images and videos of slim bodies are found on social media platforms, people who do not have a proper constitution or body shape that agrees with them, will face weight stigma, thus developing negative attitudes associated with weight, devaluation (192). A high level of body appreciation will allow counteracting ideal images of beauty on social media, by keeping a proper image of the body, respecting and valuing it (95, 96). People who value their bodies do not just describe themselves in positive terms, they value themselves for who they are, without having to relate to unrealistic standards made up by social media influencers. The results of our study indicate that exposure to social media content, more specifically, images and videos, of thin bodies, considered to be benchmarks in terms of the ideal of beauty and attractiveness, leads to the internalization of unrealistic standards regarding appearance, and the process of comparing these images of ideal bodies to the real image increases the level of dissatisfaction with one’s own body. As well as the tendency to self-devaluation, aspects that will favor the adoption of unhealthy, restrictive or emotional eating behaviors. We consider that there is an interconnection relationship between the psychological variables mentioned above, which produces effects at psychological, behavioral and health level, and these have an impact on the level of public health. Regarding the psychological implications, we will refer to the consequences felt at an emotional, cognitive and motivational level. Thus, viewing photos and videos with attractive bodies will trigger the appearance of negative emotions, such as anger, shame towards one’s own body, guilt, disgust, which can later turn into generalized anxiety or even depression (157). Regarding the cognitive plan, the negative evaluation of the body, consequently the formation of attitudes with the same valence, starts from the set of ways in which lean and fat bodies are perceived. A higher value is given to all elements related to what constitutes a slim body, and by comparison, a negative connotation to elements related to the representation of fat bodies. The onset of unhappy thoughts about one’s body turns into behavioral intention, the desire to improve appearance, to have and display a body that is worthy of consideration, appreciated by others, because it corresponds to generally valid standards for this historical stage. And subsequently, this desire, which becomes a primary need, affects how food and food decisions are valued. Food categories will be categorized according to their ability to maintain, keep a slim body, for those who have a normal body mass index, or a body mass index below average, including people with eating disorders, such as bulimia, anorexia, or those that will lead to weight loss, especially for people who have an above-average body mass index, overweight people. In a study conducted on a group of 1,200 participants, which aimed to investigate attitudes towards food according to body mass index, we notice that there are no significant differences in the category of those who had a normal body mass index compared to overweight people (193). Thus, at the behavioral level, we are witnessing an increasing tendency of people to eat because of the need to get an ideal appearance, which is based on unrealistic standards, and not having a healthy body, associated with a healthy lifestyle. The increasing number of people with an eating disorder report public health-related problems involving mental health and physiological health. Between the two there are interdependence relationships, a low level of mental health will lead to physiological disorders, and the presence of diseases produces repercussions on the human psyche. A recent study (194) presents research data confirming increasing rates of the number of eating disorders among children and adolescents, with Covid-19 pandemic making a major contribution to this phenomenon.

## 6 Conclusion

The present research highlights the role that mass media plays in developing a positive body image and healthy body appreciation, as well as in setting benchmarks associated with beauty and attractiveness standards, social representations of eating behavior, perceptions of dietary restrictions, and diets. Although social media representatives, representatives of the fit-inspiration and thin-inspiration movement (7, 195) start from the premise that they will send messages with a role in improving body shape by correcting healthy eating behavior, the effects are not as expected, so most young people and young adults tend to evaluate themselves rather negatively, comparing (196, 197) unrealistic benchmark images with the real image, restrictions and food prohibitions emerge as a way to improve the perception of body shape, to diminish the level of dissatisfaction. The results of a population study of young women indicate that a one-week break from social media leads to improved aspects of body satisfaction and body image (198, 199). Thus, we highlight the impact that the consumption of social media content has on the quality of body perceptions and body appreciation.

### 6.1 Research limits

The main limitation of the research refers to the fact that the group was predominantly made up of women, who are more open to participate in studies (200, 201) involving the disclosure of personal information (202), gender differences also imply the valence of information, men being less willing to share negative information (203) and more interested in aspects related to body shape, beauty (74). Misconceptions about eating behavior disorders, e.g., eating problems are associated with the need for attention, vanity, food choices are personal choices, and diets are part of life (204, 205). The men are more satisfied with their bodies than women, if we take into account adolescence, subsequently, with age, we witness an improvement in these aspects, only among women. Given that the participants in our study were young people and young adults, we identified a higher level of body appreciation among men than among women (85). A possible explanation could be related to the fact that mature women, with life experience, learn to focus on the qualities they have (206, 207), to the detriment of those that are exposed on social media, and men, because they are not so influenced by media content, keep the same criteria for evaluating appearance (121, 198). Thus, women are forced throughout their lives to manage the discrepancies between what social media promotes, the appearance they must have, and the one they own, the discrepancy between the ideal facet of the body and the real one. Another explanation refers to the fact that mature women have a lower level of self-objectivation, but also of sexual-objectivation, which is due to the social media content that is created for other age groups, thus, naturally decreases the pressure that mature women feel regarding appearance, the need to have a slim body to be considered valuable.

A possible limitation of the research is the specificity of the approached topic, in the sense that it limits the research area to a very specific issue for which specific variables are available. The researches that could optimally complement the results of the current approach could benefit from statistical analyses that explicitly validate the advanced model.

### 6.2 Future research directions

In order to capture the issues related to social-media pressure on the tendency to internalize standards of beauty and attractiveness of thin bodies, which leads to distortion of perception about body shape, affects the extent to which individuals appreciate their body, as well as repercussions on eating behavior, the study can be continued by expanding the group of participants, including people from other age groups, starting from childhood, but also drawing longitudinal research designs. Studies (120, 208) indicate that these aspects are evident from the first years of life, on the one hand, and their presence is then maintained, until adulthood, on the other hand. Given that the number of people suffering from anxiety and depression (2, 209, 210) is becoming greater, from increasingly young ages, and the quality of mental and physical health suffers, and the effects are associated with the quality of eating behaviors, especially the emotional one, designing a research design to evaluate the moderating role of anxiety and depression on the relationship between the perception of body shape and the presence of eating behavior disorders will allow the collection of useful results in developing strategies aimed at developing a healthy self-image by inoculating appropriate models.

### 6.3 Practical implications

The present study can be a starting point for drawing up a national strategy necessary to attract an alarm signal regarding the influence that social media has on the design of beauty and attractiveness models, as well as on the quality of eating behaviors, implicitly on mental and physical health. The outcome of the research contributes to the existing specialized literature by presenting results that highlight some of the defining characteristics for this historical stage: the strong technology and its impact on the approach of building body shape perceptions, the tendency to internalize media content, which affects the manner of relating to oneself and influences the valence of attitudes and eating behaviors.

## Data Availability

The raw data supporting the conclusions of this article will be made available by the authors, without undue reservation.
