# Supplementary material for: The healthy eating movement on social media and its psychological effects on body image
Source: Front Nutr. 2024 Dec 17;11:1474729. doi: 10.3389/fnut.2024.1474729 (PMC11685096; doi:10.3389/fnut.2024.1474729)
Supplement: Supplementary file 1 [file Data_Sheet_1.pdf]

Annex no 1

Variables from the tested model		
**Construct /Item from questionnaire**	**Scale**	**Adapted from**
**Socio-Media Pressure for a Thin Body Image (SMPTB)**	Definitely disagree Definitely agree<br>1 2 3 4 5	**Schaefer, L., et. al., (2013)**
1. I feel pressure from the media to look in better shape.<br>2. I feel pressure from the media to look thinner.<br>3. I feel pressure from the media to improve my appearance.<br>4. I feel pressure from the media to decrease my level of body fat.		
**Thin Body Image Standard Internalization (TBISI)**	Definitely disagree Definitely agree<br>1 2 3 4 5	**Schaefer, L., et al., (2017)**
1. It is important for me to look athletic.<br>2. I think a lot about looking muscular.<br>3. I want my body to look very thin.<br>4. I want my body to look like it has little fat.<br>5. I think a lot about looking thin.		
**The Body Shape Perception (BSP)**	Never Always<br>1 2 3 4 5 6	**Evans, C., et al. (1993)**
1. Have you been so worried about your shape you have been feeling you ought to diet?<br>2. Have you been afraid that you might become fat (or fatter)?<br>3. Has feeling full (e.g. after eating a large meal) made you feel fat?<br>4. Have you notice the shape of other women and felt that your own shape compared unfavorably?<br>5. Has thinking about your shape interfered with your ability to concentrate (e.g. while watching television, reading, listening to conversations)?<br>6. Has being naked, such as when taking a bath, made you feel fat?<br>7. Have you imagined cutting off fleshy areas of your body?<br>8. Have you not gone out to social occasions (e.g. parties) because you have felt bad about your shape?<br>9. Have you felt excessively large and round?<br>10. Have you thought that you are in the shape you are because you lack self-control?<br>11. Have you worried about other people seeing rolls of fat around your waist or stomach?<br>12. When in company have your worried about taking up too much room (e.g. sitting on a sofa, or a bus seat).<br>13. Has seeing your reflection (e.g. in a mirror or shop window) made you feel bad about your shape?<br>14. Have you pinched areas of your body to see how much fat there is?<br>15. Have you avoided situations where people could see your body (e.g. communal changing rooms or swimming baths)?<br>16. Have you been particularly self-conscious about your shape when in the company of other people?		
**Body Appreciation (BA)**	Never Always<br>1 2 3 4 5	**Tylka, T. L., Wood-Barcalow, N. L, (2015)**
1. I respect my body.<br>2. I feel good about my body.<br>3. I feel that my body has at least some good qualities.<br>4. I take a positive attitude towards my body.<br>5. I am attentive to my body's needs.		

6. I feel love for my body.<br>7. I appreciate the different and unique characteristics of my body.<br>8. My behavior reveals my positive attitude toward my body; for example, I walk holding my head high and smiling.<br>9. I am comfortable in my body.<br>10. I feel like I am beautiful even if I am different from media images of attractive people (e.g., models, actresses/actors).		
**Restrained Eating Behavior (REB)**	Never Very often<br>1 2 3 4 5	**Van Strien, T., et al., (1986)**
1. If you have put on weight, do you eat less than you usually do?<br>2. Do you try to eat less at mealtimes than you would like to eat?<br>3. How often do you refuse food or drink offered because you are concerned about your weight?<br>4. Do you watch exactly what you eat?<br>5. Do you deliberately eat foods that are slimming?<br>6. When you have eaten too much, do you eat less than usual the following days?<br>7. Do you deliberately eat less in order not to become heavier?<br>8. How often do you try not to eat between meals because you are watching your weight?<br>9. How often in the evening do you try not to eat because you are watching your weight?<br>10. Do you take into account your weight with what you eat?		
**Emotional Eating Behavior (EEB)**	Never Very often<br>1 2 3 4 5	**Van Strien, T., et al. (1986)**
1. Do you have the desire to eat when you are irritated?<br>2. Do you have a desire to eat when you have nothing to do?<br>3. Do you have a desire to eat when you are depressed or discouraged?<br>4. Do you have a desire to eat when you are feeling lonely?<br>5. Do you have a desire to eat when somebody lets you down?<br>6. Do you have a desire to eat when you are cross?<br>7. Do you have a desire to eat when you are approaching something unpleasant to happen?<br>8. Do you get the desire to eat when you are anxious, worried or tense?<br>9. Do you have a desire to eat when things are going against you or when things have gone wrong?<br>10. Do you have a desire to eat when you are frightened?<br>11. Do you have a desire to eat when you are disappointed?<br>12. Do you have a desire to eat when you are emotionally upset?<br>13. Do you have a desire to eat when you are bored or restless?		
